# Coordinating Between Medical Professions’ Tasks to Optimize Sub-Saharan Health Systems: A Response to Recent Commentaries

**DOI:** 10.15171/ijhpm.2016.142

**Published:** 2016-10-30

**Authors:** Nir Eyal, Corrado Cancedda, Samia A. Hurst, Patrick Kyamanywa

**Affiliations:** ^1^Department of Global Health and Population, Harvard TH Chan School of Public Health, Boston, MA, USA.; ^2^Division of Global Health Equity, Brigham and Women’s Hospital, and Department of Global Health and Social Medicine, Harvard Medical School, Boston, MA, USA.; ^3^Institute for Ethics, History, and the Humanities, Faculty of Medicine, Geneva University, Geneva, Switzerland.; ^4^School of Health Sciences, Kampala International University, Kampala, Uganda.

We are grateful that our perspective^1^ received commentary from leading experts on African human resources for health. All endorse and several quote our central suggestion that the “development in [non-physician clinician] deployment should unfold in parallel with strategic rethinking of the role of physicians and with critical innovations in physicians’ education and in-service training.”

Given the respondents’ expertise and number, this symposium can perhaps be seen as an informal consensus statement in support of greater coordination between different medical professions’ role definitions and training benchmarks. In sub-Saharan Africa, non-physician clinicians (NPCs) have existed for many years,^2-4^ and they and other non-traditional professionals are increasingly assuming the bulk of clinical tasks. Now is the time both to streamline their roles as providers of clinical services and to integrate them with the rest of the health system. That affects physicians as well. System-level coordination, necessary for physicians in primary care roles with other physicians,^5^ is a broader necessity—for everyone, in all roles, including for physicians vis-à-vis NPCs.

Commentators also agree on sub-Saharan Africa’s pressing need for task delegation to NPCs and other associate health workers, for additional rural externships and rurally-focused curricula, and for higher budgets towards incentivizing physicians’ and NPCs’ rural deployment. The present response focuses on four potential areas of disagreement.

## What is an Non-physician Clinician?

Some respondents^4,6^ criticize our definition of NPCs, originally by Mullan and Frehywot, according to which NPCs are “health workers who have fewer clinical skills than physicians but more than nurses.”^7^ On any definition by clinical skill, inter-individual skill variance may count as variance in professional–affiliation. To prevent that quirk, one might try to define the skill level as the mean level among practitioners in the relevant country. But that would make the definition country-relative, because mean skill levels vary between countries (eg, because the allowed scope of service by professional regulatory bodies varies). A problem with any definition in terms of skill *level* is that it compels us to pronounce on which profession has the highest overall “level”—although each has some unique skills. We concur, therefore, that a different definition, perhaps based on the different skill levels *traditionally ascribed* to each profession, or on their* tasks and responsibilities* instead of their skills, may be better.

## Should Practitioners’ Independence and Scope of Work Be Fixed Individually, by Their Profession, or Otherwise?

Dovlo et al discuss the “delineation of tasks between physicians and NPCs … at the same service delivery point.”^4^ They recommend that “scopes and the relative independence of practice of both NPCs and physicians shall depend on the circumstances and experience of each cadre type. An NPC practicing in a remote inaccessible area that has significant experience… may need less oversight than newly qualified physicians….”^4^ At the other extreme, Gottlieb Monekosso re-affirms the World Health Organization (WHO) earlier definition of scopes as uniform across professions.^2^ Should tasks be delineated per individual practitioner and her individual skills, or per profession?

The attraction of individualized task delineation is nuance, as well as more equal opportunities, unbound by professional affiliation. But, there is something to be said for profession-wide standardization as well. Standardization facilitates coordination, harmonizes performance evaluation, and, by setting uniform expectations, may preempt personal offense and tension. Moreover, coupling standard training for NPCs with special fit for resource-poor settings may have helped limit NPCs’ attrition to the private sector and foreign countries. An intriguing intermediate measure is proposed by Sidat: adjusting the scope of work to the likely first job after school, for either physicians or NPCs. His intriguing proposal defines role definitions differently for areas where the first job is likely to be in primary care than for ones where the first job is likely to be at district hospitals.^8^

Finding the correct answer to these questions may require health system studies that compare different approaches empirically.^9^

## Should Managerial and/or Educational Roles Be Reserved to Physicians Only?

Some writers warn against assuming that physicians should occupy all managerial roles,^4,10^ or all educational ones.^4,10^ They suggest that NPCs or non-medical staff could also fill those roles.

Systems thinking about role definitions for different professions remains open to this thought. What we said applies only to those areas where, despite attempts to shift managerial and/or educational tasks, physicians and their longer training or professional aura have been shown to remain irreplaceable. A physician managing a small team may turn out on rigorous examination to bring authority and smooth coordination that other professionals could not. A physician may turn out to prove essential in specialist roles. But there may be individual exceptions. In Uganda for example, senior NPCs are also assigned managerial, educational, and supervisory roles in the lower tiers of the health system. Again, empirical studies could be a good way to move forward.

## Does the Increasing Clinical Burden Permit us to Leave Physicians Untrained for Their Non-clinical Roles?

Dovlo et al criticize our proposal “to train all physicians with managerial, mentoring, and supervisory skills, whether they will need them or not.” They deem that proposal blind to medical students’ already burdened curricula, given the “double burden” of communicable and noncommunicable disease and new attention to “lifestyle and behavioral conditions.” Not all graduates, they emphasize, will require these non-clinical skills.^4^ Binagwaho et al declare, more conditionally, “we, therefore, advocate that education in non-clinical knowledge must only be put in place if it improves clinical medical skills and education, in lieu of reducing or replacing it.”^10^

Our response is to insist that every professional, physicians included, should be equipped with any managerial and mentorship skills necessary for them, from the system’s viewpoint. This position is supported by literature and curriculum reviews that advocate for the inclusion of health service management modules. Where NPCs provide the mainstay of clinical response, the skills necessary for optimizing system-level results in collaboration with them only increase.

While there is always scope for some diversity in doctors’ training, most students will need those nonclinical skills if the health system is to remain responsive to patients’ needs rather than to aspirations that doctors developed before the change occurred. Outside the special, nonreplicable context of intensely funded and human resource rich projects, difficult triage on what skills to prioritize in training is unavoidable.

Regarding attention to lifestyle and behavioral conditions in the clinic, we certainly hope that clinical tasks and knowledge about behavioral science will increase in the future; in our view, however, that will largely affect frontline NPCs’ roles and training, not doctors.’

Some respondents point out additional non-clinical skills that have become necessary: mHealth and eHealth skills; the ability to “mobilize and coordinate with other stakeholders in the community [and] aid agencies”^6^; in-practice training of generalist providers in primary healthcare^11^; and the willingness to accept further training from highly experienced NPCs.^4^ These possibilities merit further exploration.

## Conclusion

We wanted to end by pointing to open questions about coordinating between the scopes of work of physicians and those of other health professionals in sub-Saharan Africa, as well as with a further note of appreciation for our respondents. The best way to do both is to refer readers to the excellent list of open questions in commentators Dussault and Cobb’s section “A challenging policy agenda.”^6^

## Ethical issues

Not applicable.

## Competing interests

Authors declare that they have no competing interests.

## Authors’ contributions

PK, SAH, CC, and NE were authors of the article whose commentary is being responded to. NE drafted the response and CC, SAH, and PK read, edited, and added to the development of the draft.

## Authors’ affiliations

^1^Department of Global Health and Population, Harvard TH Chan School of Public Health, Boston, MA, USA. ^2^Division of Global Health Equity, Brigham and Women’s Hospital, and Department of Global Health and Social Medicine, Harvard Medical School, Boston, MA, USA. ^3^Institute for Ethics, History, and the Humanities, Faculty of Medicine, Geneva University, Geneva, Switzerland. ^4^School of Health Sciences, Kampala International University, Kampala, Uganda.
